# Characterization of novel microsatellite markers in *Musa acuminata *subsp. *burmannicoides*, var. Calcutta 4

**DOI:** 10.1186/1756-0500-3-148

**Published:** 2010-05-27

**Authors:** Robert NG Miller, Marco AN Passos, Natalia NP Menezes, Manoel T Souza, Marcos M do Carmo Costa, Vânia C Rennó Azevedo, Edson P Amorim, Georgios J Pappas, Ana Y Ciampi

**Affiliations:** 1 Universidade de Brasília, Campus Universitário Darcy Ribeiro, Instituto de Ciências Biológicas, Departamento de Biologia Celular, Asa Norte, Brasília, Brazil; 2 Universidade Católica de Brasília, SGAN 916, Módulo B, Brasília, Brazil; 3 EMBRAPA Agroenergia, Parque Estação Biológica - PqEB - Av. W3 Norte, Brasília, Brazil; 4 EMBRAPA Recursos Genéticos e Biotecnologia, Parque Estação Biológica - PqEB - Av. W5 Norte, Brasília, Brazil; 5 EMBRAPA Mandioca e Fruticultura Tropical, Rua Embrapa, Cruz das Almas, Brazil

## Abstract

**Background:**

Banana is a nutritionally important crop across tropical and sub-tropical countries in sub-Saharan Africa, Central and South America and Asia. Although cultivars have evolved from diploid, triploid and tetraploid wild Asian species of *Musa acuminata *(A genome) and *Musa balbisiana *(B genome), many of today's commercial cultivars are sterile triploids or diploids, with fruit developing via parthenocarpy. As a result of restricted genetic variation, improvement has been limited, resulting in a crop frequently lacking resistance to pests and disease. Considering the importance of molecular tools to facilitate development of disease resistant genotypes, the objectives of this study were to develop polymorphic microsatellite markers from BAC clone sequences for *M. acuminata *subsp. *burmannicoides*, var. Calcutta 4. This wild diploid species is used as a donor cultivar in breeding programs as a source of resistance to diverse biotic stresses.

**Findings:**

Microsatellite sequences were identified from five Calcutta 4 BAC consensi datasets. Specific primers were designed for 41 loci. Isolated di-nucleotide repeat motifs were the most abundant, followed by tri-nucleotides. From 33 tested loci, 20 displayed polymorphism when screened across 21 diploid *M. acuminata *accessions, contrasting in resistance to Sigatoka diseases. The number of alleles per SSR locus ranged from two to four, with a total of 56. Six repeat classes were identified, with di-nucleotides the most abundant. Expected heterozygosity values for polymorphic markers ranged from 0.31 to 0.75.

**Conclusions:**

This is the first report identifying polymorphic microsatellite markers from *M. acuminata *subsp. *burmannicoides*, var. Calcutta 4 across accessions contrasting in resistance to Sigatoka diseases. These BAC-derived polymorphic microsatellite markers are a useful resource for banana, applicable for genetic map development, germplasm characterization, evolutionary studies and marker assisted selection for traits.

## Background

Commercial banana varieties, which are derived from intraspecific crosses within *Musa acuminata *Colla, together with interspecific hybrid development with *Musa balbisiana *Colla, are cultivated mostly by smallholder farmers, across over 120 countries in different tropical and sub-tropical environments. As an inexpensive starch source, banana is also rich in fibre, minerals and vitamins. Although an important food commodity in developing countries in terms of production value after rice, wheat and maize, genetic improvement has been limited. In wild bananas, sexual recombination results in viable seed. However, the majority of today's commercial cultivars are sterile A and B genome-containing triploids, with seedless fruit development occurring via parthenocarpy, partly as a result of translocations [1]. Conventional breeding in *Musa *diploids and triploids is also hampered as a result of a low number or complete absence of seeds, caused by either a lack of viable pollen, or inefficient pollinating insects. As many cultivars are evolving asexually via vegetative micropropagation or suckers, their genetic base is narrow, resulting in crops lacking resistance to pests and disease. Given the large scale global consumption of susceptible genotypes such as the sterile triploids of the *M. acuminata *Cavendish cultivar group, global *Musa *production faces threats by fungal, bacterial and viral pathogens and a number of pests, with greatest disease losses today caused by the fungal pathogens *Mycosphaerella fijiensis*, causal organism of black Sigatoka disease, and *Fusarium oxysporum *f. sp. *cubense *Tropical Race 4, which causes Fusarium wilt. For these reasons, molecular tools for the development of disease resistant genotypes are of paramount importance for the *Musa *industry.

Highly variable microsatellites or simple sequence repeat loci (SSRs), are abundant, randomly dispersed, locus specific, codominant and multi-allelic markers, which are composed of core repeat sequences of, for example, di- to penta-nucleotides, repeated in tandem. Their application in *Musa *has included genotyping [2-4], *Musa *evolution and taxonomy [5], and linkage map saturation [1]. Potential also exists in marker assisted selection (MAS), upon identification of SSRs for gene loci co-localizing with quantitative trait loci (QTLs) for desirable traits. To date, several hundred SSR markers have been developed from *M. acuminata *and *M. balbisiana *material [5,2-8]. In comparison with other crop species, however, the total number available for genetic analyses remains limited, given that alleles can be absent or monomorphic when applied across cultivars.

We report the development of novel SSR markers from sequenced BAC clones in *M. acuminata *Calcutta 4. This wild diploid species is resistant to numerous fungal and bacterial pathogens, as well as nematodes. Given its' potential as a source of exploitable genes, this cultivar is widely employed as a donor species in banana breeding programs [9]. Polymorphic loci were identified when tested across 21 potential parental diploid *M. acuminata *individuals contrasting in resistance to Sigatoka diseases caused by the ascomycete fungi *M. fijiensis *and *Mycosphaerella musicola. *Such BAC-derived markers are potentially advantageous in that polymorphism can not only be greater than that observed using EST-derived SSRs [10], but subsequent mapping also allows anchoring of individual BAC clones of interest to genetic maps.

## Results

The sequences of five *Musa *BAC clones were subjected to a computational pipeline targeting perfect SSRs with periodicities ranging from two to ten nucleotides, and an overall length of 12 bases. In total, 41 SSRs were identified comprising six repeat classes. Di-nucleotide repeats are the most abundant (46.34%) class, followed by tri- (29.26%), tetra- (12.19%), penta- (7.31%), hexa- (2.43%) and nona-nucleotide repeats (2.43%). The most abundant dinucleotide repeat motifs isolated were AG, AT, CT, and TA (7.31% each). By contrast, all tri-nucleotide motifs were equal in abundance (7.31% each). Generally, the shorter the nucleotide core sequence, the greater were the number of repeats observed, with an average of 12.2 repeats for di-nucleotide motifs, 5.8 for tri, 3.6 for tetra, 3 for penta, 3 for hexa, and 3 for nona-nucleotide motifs. A summary of all designed primer sequences, SSR motifs, theoretical annealing temperature, and expected product size is provided for the 41 loci identified where primers could be designed [Additional file 1]. Twenty out of 33 tested primer pairs reproducibly amplified polymorphic PCR products across the *Musa *accessions, with allelic patterns under optimized primer conditions given in Table 1. Di-nucleotide repeats were the most abundant polymorphic group, followed by tri, penta and tetra-nucleotides. From a total of 56 scored alleles, the number of polymorphic alleles ranged from two to four, with an average of 2.8 alleles per locus. Heterozygosity values were calculated using GDA [11] and FSTAT [12], with expected values ranging from 0.31 to 0.75. Thirteen loci (MABN 09, MABN 12, MABN 14, MABN 16, MABN 18, MABN 21, MABN 24, MABN 31, MABN 33, MABN 37, MABN 38, MABN 39, and MABN 40) were monomorphic in *M. acuminata *accessions. Twelve loci showed departure from Hardy-Weinberg expectations (P < 0.05 using Fisher's exact test probability [P < 0.05] based on 2000 shufflings), possibly as a result of sampling, chromosomal inversions or null alleles. Phenomena potentially responsible for null alleles include point mutations and sequence divergence in primer annealing sites, or preferential allele amplification during PCR. In testing for linkage disequilibrium (LD) (FSTAT P < 0.01 with Bonferroni correction), no disequilibrium was detected among the loci pairwise combinations. PIC values for allelic diversity ranged from 0.258 to 0.681.

**Table 1:** Characteristics of microsatellite loci isolated from *M. acuminata *Calcutta 4 and polymorphic across 21 *M. acuminata *accessions.

Locus name	BAC consensus sequence ID	**GenBank Accession no**.	Repeat Array	Primer Sequence (5' - 3')	Obtained allele size range (bp)	**T**_**m **_**(**^**o**^**C) used**	***N***_***a***_	***H***_***E***_	***H***_***O***_	*HWE P *value	PIC
MABN01	MA4_008L021	AC186748	(AG)12	F: CCACTGAAGCTGAAAGGAGG	500-540	56	3	0.667828	0.875000	0.021000*	0.577
				R: GGATTGTAGGTGACGGGAGA		56					
MABN03	MA4_008L021	AC186748	(TG)10	F: TGGTTGTATGTTTGCTGGGA	500-545	60	3	0.593590	0.850000	0.013500*	0.504
				R: CAAAGTGCTGGCATGAGAAA		60					
MABN06	MA4_008L021	AC186748	(ATAC)3	F: GCAACCATCAACCAAAAACC	344-360	58	3	0.444872	0.200000	0.013500*	0.365
				R: TTTGCAAGAAAATCGTGCTG		58					
MABN07	MA4_008L021	AC186748	(ATA)6	F: TTTTGATCATCATATGGGTCG	500-540	60	2	0.344948	0.428571	0.512000	0.258
				R: AGAGGGAGAGCCAAAGTGGT		60					
MABN08	MA4_008L021	AC186748	(GA)13	F: TTACCGTAAACGGAGCCAAC	260-290	58	3	0.637631	1.000000	0.000000*	0.544
				R: GAAATCGAGGAAAACCGACA		58					
MABN13	MA4_111B014	AC186954	(CA)6	F: CCTCAACGAAGCATACAGCA	210-240	58	2	0.450980	0.647059	0.106500	0.351
				R: CAGTCTGGGCTGACACAGAA		58					
MABN15	MA4_111B014	AC186954	(ATTTT)3	F: CCAACTTCCATTTGGCTTTT	490-520	58	2	0.315912	0.380952	1.000000	0.258
				R: CGCAGGCGACTTCTTACAGT		58					
MABN17	MA4_111B014	AC186954	(TCT)14	F: CCCATGCAACTACAACAACG	200-245	60	4	0.732804	1.000000	0.125000	0.659
				R: GGAACCACGTGTCCTGATCT		60					
MABN19	MA4_106O017	AC186747	(TTTAT)3	F: CTCCACCGCTGCAAATTAT	330-380	60	4	0.750794	0.944444	0.003000*	0.681
				R: TTCATTTGATTGGAAAGTGGAA		60					
MABN20	MA4_106O017	AC186747	(AC)7	F: AAGAAGTGCAACAGATGGGC	344-380	56	3	0.537179	0.550000	0.727500	0.454
				R: GCCAAAGGAATCATGCTGTT		56					
MABN22	MA4_106O017	AC186747	(AG)6	F: GTCGCAGAGATCAAGGAACC	490-510	58	2	0.507549	0.619048	0.392000	0.373
				R: GGACCTCCTATGTTTGCTGC		58					
MABN23	MA4_106O017	AC186747	(TTA)4	F: TCGATCATTTGGCATCACAT	350-500	60	4	0.723577	0.952381	0.015500*	0.641
				R: CCAGGTAGCGAAGACGAGAC		60					
MABN25	MA4_106O017	AC186747	(TAT)9	F: TTTCATGATTTGAGGAGCCC	380-410	58	2	0.462304	0.684211	0.049500*	0.348
				R: CCCCACAAGTATGTTCCCAC		58					
MABN26	MA4_106O017	AC186747	(CT)24	F: GTGGGAACATACTTGTGGGG	375-395	58	2	0.493612	0.047619	0.000000*	0.359
				R: ACGGAAAACCACAAGCAATC		58					
MABN27	MA4_106O017	AC186747	(GAA)4	F: GGATGCAAAGACGGACAAAT	470-520	58	3	0.667828	0.714286	0.000000*	0.575
				R: TAATGGCTTTGCAACTGCTG		58					
MABN28	MA4_106O017	AC186747	(GA)23	F: TGGAGGTCTCAACCAAAACC	390-410	60	2	0.480769	0.550000	0.639500	0.367
				R: AGATTGGCTACTGTGGGTGG		60					
MABN29	MA4_106O017	AC186747	(GAT)5	F: ACCAGCCACTGGAATCAAAC	350-385	60	3	0.600000	0.866667	0.069000	0.506
				R: GTCTGCTGAAGAGCCAAACC		60					
MABN30	MA4_106O017	AC186747	(ATTTT)3	F: CAGCCGTTGATGTTCAAATG	360-380	60	2	0.387097	1.000000	0.000500*	0.321
				R: CGTTACGGTGGATCGTCTTT		60					
MABN34	MA4_106O017	AC186747	(CT)18	F: TAGGTGAGAATGGGACGGAG	330-355	58	3	0.661451	0.368421	0.000000*	0.571
				R: CAGTAGCAGCAACCTGGTGA		58					
MABN35	MA4_106O017	AC186747	(CT)14	F: CTGTCACCAGGTTGCTGCTA	270-320	56	4	0.664103	0.450000	0.005500*	0.569
				R: CTTCCTTGGACCTTTCATCG		56					

Tm, annealing temperature used; Na, number of alleles per locus observed; H_E_, expected heterozygosity under Hardy-Weinberg expectations; H_O_, observed heterozygosity; H-W, P value for deviation from Hardy-Weinberg equilibrium, with *significant departure (P < 0.05) from HW equilibrium; PIC, Polymorphism Information Content

## Discussion

This is the first report identifying polymorphic microsatellite markers from *M. acuminata *Calcutta 4 across accessions contrasting in resistance to Sigatoka diseases. The availability of these molecular tools will contribute towards development of genetic maps with high marker density, derived from segregant populations for agronomically important traits, and offering potential for downstream application in MAS. Concerted efforts are currently underway by a number of *Musa *breeding groups for development of segregant mapping populations [13,14].

Also, given difficulties in development of populations in *Musa *with sufficient numbers of individuals for high resolution mapping, LD mapping has been proposed as an alternative route for identifying genes for traits of interest in *Musa *[15]. As such an approach requires both hundreds of plant accessions and thousands of markers, the new microsatellite markers characterized in this study can serve as candidates for such work. Our markers are also a resource for characterizing diversity in wild species, cultivars and landraces deposited in genebanks, and for inferring phylogenetic relationships in *Musa*.

Finally, considering the increasing availability of genomic resources for *M. acuminata *Calcutta 4, such as BAC libraries [16], EST data sets [17] and candidate disease resistance gene sequences [18], in the context of available next generation sequencing technologies, identification of genes and markers for desirable traits such as resistance to biotic stress will no doubt accelerate considerably in the near future.

## Conclusion

In this study 41 new microsatellite markers were developed for *M. acuminata*, of which 20 displayed reasonable polymorphism when screened across 21 diploid individuals contrasting in resistance to Sigatoka diseases. Polymorphic markers detected an average of 2.8 alleles per locus, with PIC values ranging from 0.258 to 0.681. The results also provided some information on repeat class nature and abundance.

## Methods

Data for SSR identification was derived from genomic data (shotgun-sequenced BAC clones from a *M. acuminata *Calcutta 4 BAC library) [16,19]. A computational search over five BAC consensi datasets [GenBank:AC186748, AC186749, AC186954, AC186747 and AC186750] was performed to locate SSRs with at least two repeating units spanning more than 10 bases, using the program Mreps [20]. Primers flanking microsatellite loci were designed using the program PRIMER3 [21].

From 41 loci identified where primers could be designed, 33 primer pairs were tested for polymorphism. Twenty one diploid (AA) *M. acuminata *accessions, contrasting in resistance to Sigatoka diseases, and potential parentals for genetic map construction, were used to characterize microsatellite loci. Genomic DNA was extracted from the Black Sigatoka-resistant *M. acuminata *accessions Calcutta 4, Lidi, 0323-03, SH32-63, 1304-06 and 0116-01; Black Sigatoka-susceptible accessions Pisang Berlin and Niyarma Yik; Yellow Sigatoka-resistant accessions Calcutta 4, Burmanica, Microcarpa, Lidi, 0323-03, 1304-06, 1741-01, 9179-03, 0116-01, 1318-01 and 4279-06; and Yellow Sigatoka-susceptible accessions Raja Uter, Tjau Lagada, F2P2, Khai Nai On, Pisang Berlin, Niyarma Yik, Sowmuk, Jaribuaya and SH32-63. Each PCR reaction was carried out in a 13 μl volume, containing 3 ng of template genomic DNA, 2.5 mM MgCl2, 0.2 mM dNTPs, 0.5 μM of each primer, 1.25 U of Taq polymerase, and 1 × PCR buffer (Invitrogen). Amplifications were conducted on a PTC-100 thermocycler (MJ Research), with temperature cycling conducted as follows: initial denaturation at 94°C for 5 min; 29 cycles of 94°C for 1 min, specific primer annealing temperature for 1 min, and extension at 72°C for 1 min; plus an extra elongation period of 7 min at 72°C. Following amplification, PCR products were initially electrophoresed in 3.5% agarose gels run in 1 × TBE buffer, in order to check amplicon size and PCR specificity. Allele sizes were estimated against 10-bp ladder molecular size standards (Invitrogen) on denaturing 6% polyacrylamide gels using 7 m urea, with PCR products visualized by silver staining according to standard protocols. The degree of polymorphism per locus was calculated using GDA software, version 1.2 [11].

## Competing interests

The authors declare that they have no competing interests.

## Authors' contributions

RNGM participated in the design and implementation of the study, and drafted the manuscript. MANP participated in microsatellite marker validation and data analysis. NNPM participated in microsatellite marker validation. MTSJ conceived the study and participated in implementation of the project. MMCC carried out computational searches for microsatellite identification and designed flanking primers. VCRA participated in data analysis. EPA supervised microsatellite marker validation and editing of the manuscript. GJPJ participated in computational searches for microsatellite identification and primer design, and editing of the manuscript. AYC conceived the study, supervised microsatellite marker validation and data analysis, and commented on the manuscript. All authors read and approved the final manuscript.

## Supplementary Material

Additional file 1 **Summary of all designed primer sequences, SSR repeat motif, theoretical annealing temperature (Tm), and expected product size**. A Microsoft Excel table containing a summary of all the designed primer sequences, together with SSR repeat motif, theoretical annealing temperature (Tm), and expected product size Click here for file
